# Chronic Encapsulated Sclerosing Peritonitis: A Case Report

**DOI:** 10.7759/cureus.58003

**Published:** 2024-04-10

**Authors:** Luis Gerardo Luna León, Julian Vargas Flores, Pabel Rubén Carbajal Cabrera, Yunuen Ailyn Morales Tercero, Hugo Fernando Narvaez Gonzalez

**Affiliations:** 1 Surgery, Hospital Regional “General Ignacio Zaragoza”, ISSSTE, Ciudad de México, MEX

**Keywords:** treatment, surgery, liver failure, chronic peritonitis, intestinal obstruction

## Abstract

Chronic encapsulated sclerosing peritonitis (CESP) is a very rare cause of intestinal obstruction that has been associated with certain chronic conditions. It is characterized by the thickening of the peritoneum, generating a membrane that covers the intestinal loops and prevents their adequate mobilization. Most cases present as a surgical emergency, leading to a diagnosis during surgery; however, imaging studies can support the pre-surgical diagnosis. Treatment is based on the clinical context of the patient, based on medical management with corticosteroids in a stable patient, or surgical management when it presents as an acute complication. The morbidity and mortality associated with this condition are high and epidemiological data are scarce. There is still a lack of studies to describe the associated demographic data, diagnosis, and treatment.

## Introduction

Chronic encapsulated sclerosing peritonitis (CESP) or “Abdominal cocoon syndrome”, is a rare condition characterized by chronic inflammation of the peritoneum that generates an encapsulation of the loops of the small intestine with a fibrous membrane formed of collagen [1]. According to the Peritoneal Dialysis Society, it is defined as a syndrome that occurs continuously, intermittently, or repeatedly with symptoms of intestinal obstruction caused by adhesions of a diffusely thickened peritoneum [2]. Sometimes it involves the stomach, colon, liver, and spleen, causing intestinal occlusion at different levels [2,3]. It is a very rare disease that presents in the patient with a wide variety of symptoms and clinical signs. The most common clinical presentation is an intestinal obstruction.

## Case presentation

**Figure 1:**
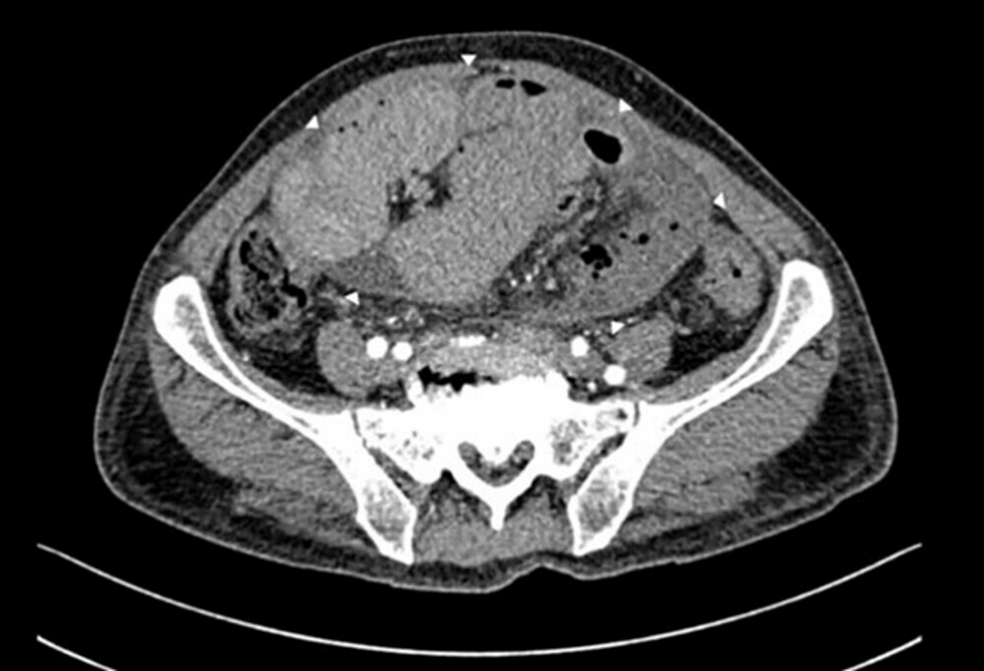
Abdominal contrast-enhanced computed tomography in axial section. Dilation of the intestinal loops with free abdominal fluid is observed. Arrowheads show thickening of the peritoneum, which surrounds and encapsulates the intestinal loops.

**Figure 2:**
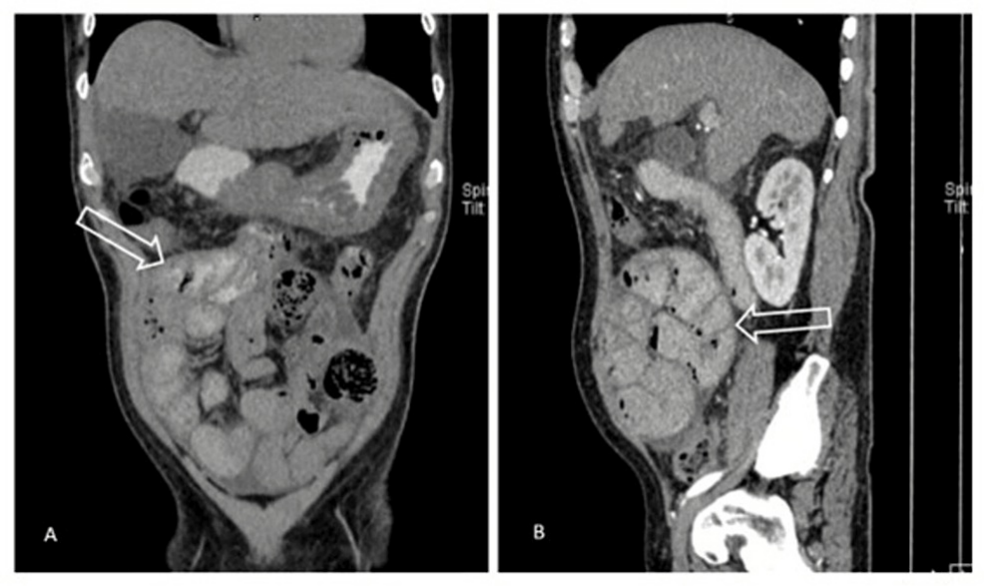
Abdominal contrast-enhanced computed tomography in coronal (A) and sagittal (B) sections. Arrows showing intestinal loops surrounded by a thick membrane known as the “cauliflower sign” characteristic of the disease.

A 57-year-old male patient presented to the emergency room with a significant history of type 2 diabetes under medical treatment and intense alcoholism since the age of 18. He had a recent diagnosis of liver failure requiring previous hospitalizations in the last six months due to bleeding from the upper digestive tract and hepatic encephalopathy. His clinical condition began with generalized colicky abdominal pain associated with nausea and vomiting of intestinal contents with an absence of evacuations for 24 hours. An evaluation by the general surgery service revealed peritoneal irritation during abdominal exploration. An abdominal CT showed abdominal fluid and free air associated with dilation of intestinal loops compatible with intestinal obstruction associated with thickening of the peritoneum surrounding the intestinal loops (Figures 1, 2).

**Figure 3:**
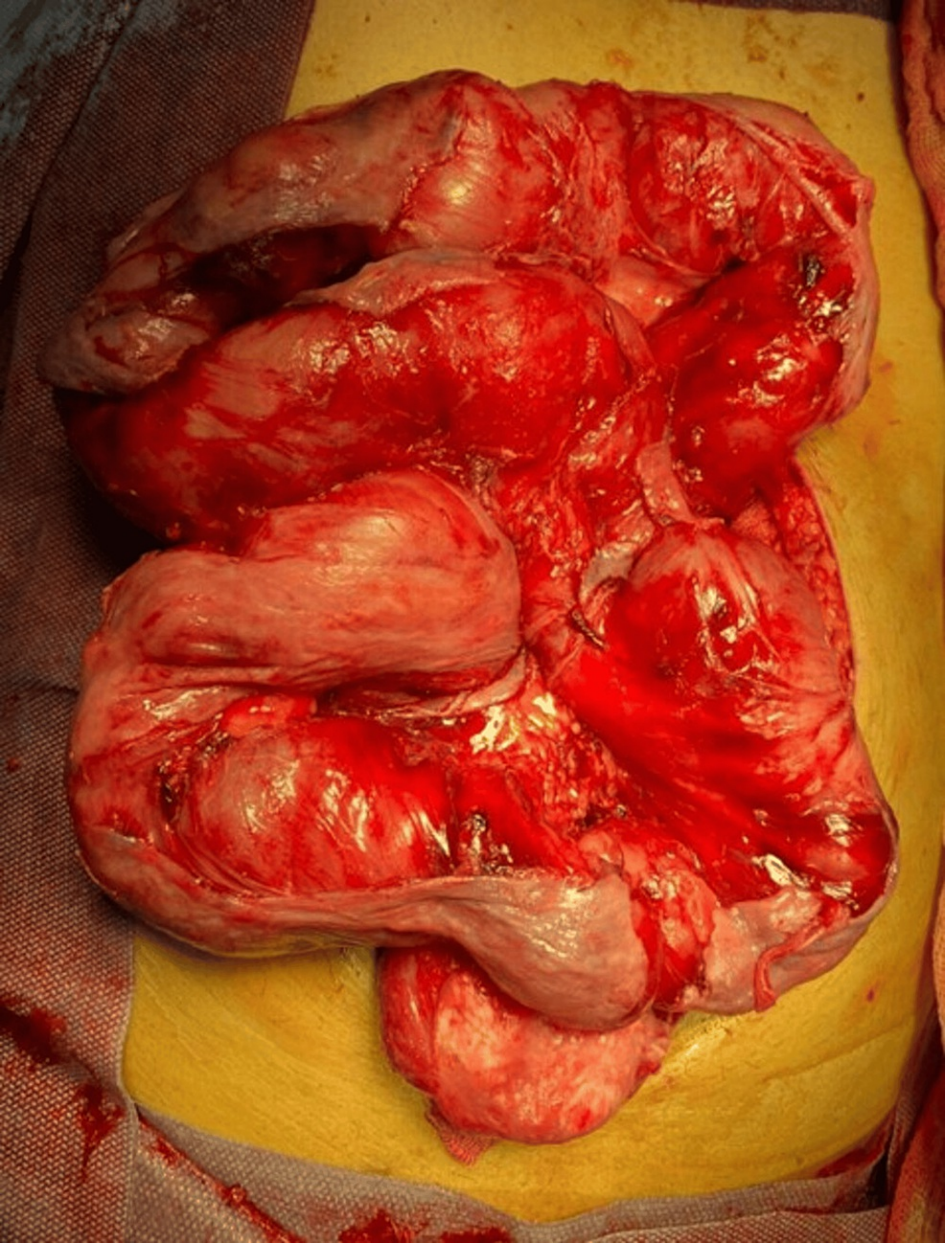
Intestinal loops covered by a fibrous layer of peritoneum on its entire surface. The dissection areas are observed with exposure of the serosa and the fiber sheath, comprising the loops of intestine.

Due to the clinical and imaging findings, he was taken to the OR to perform abdominal emergency surgery. During exploratory laparotomy, free intestinal fluid with intestinal loops completely covered by a firmly adhered fibrous membrane (Figure 3) with the absence of peristalsis was observed.

**Figure 4:**
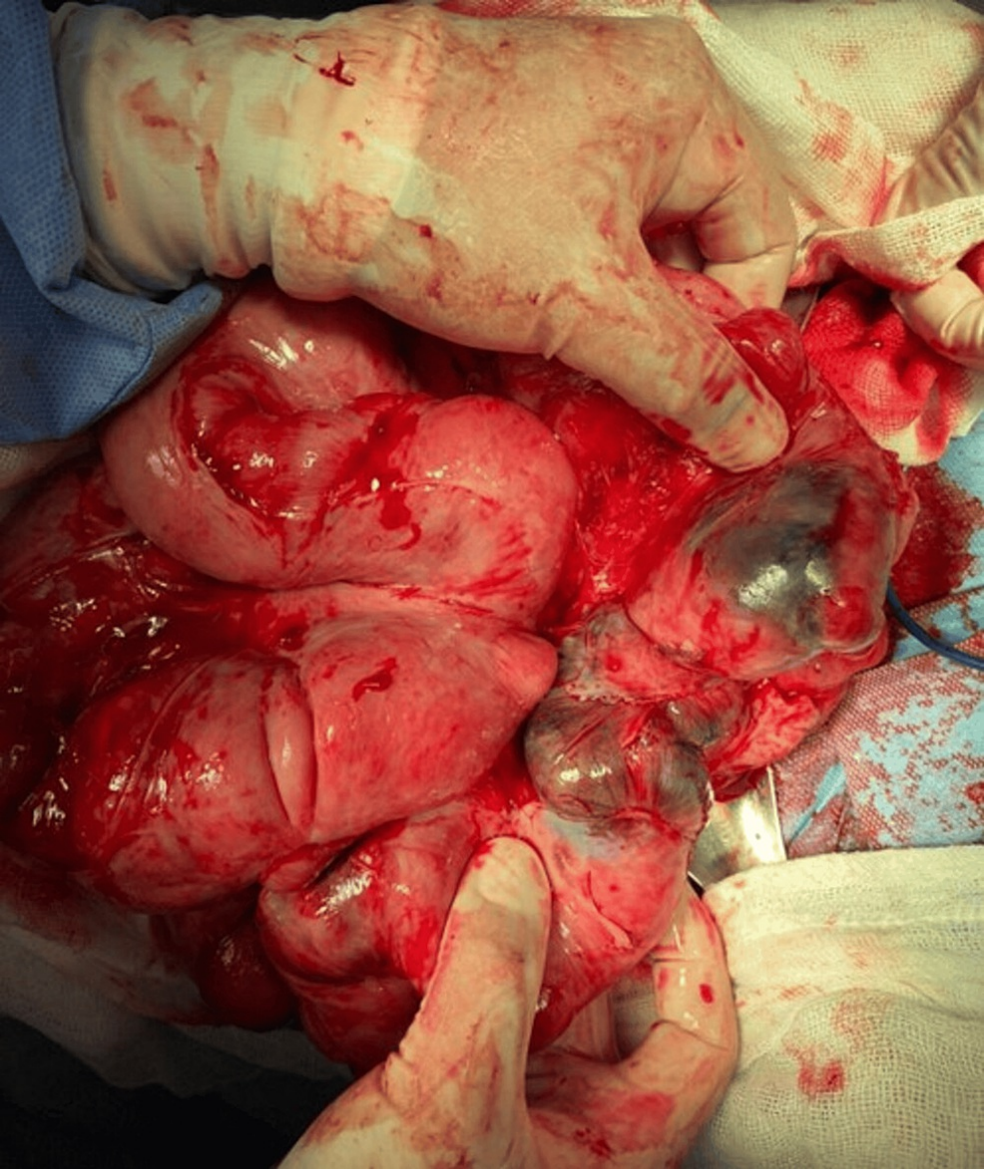
Intestinal involvement with necrosis of the intestinal wall. Exposure of area of intestinal necrosis during dissection of the fibrous layer of peritoneum.

Our team performed blunt dissection of dense membranes until exposing the serosa of the intestinal loops showing peristaltic movements. At 50 cm from the ileocecal valve, intestinal necrosis and perforation were found, compromising approximately 40 cm of the small intestine (Figure 4). Intestinal resection and mechanical anastomosis were performed.

**Figure 5:**
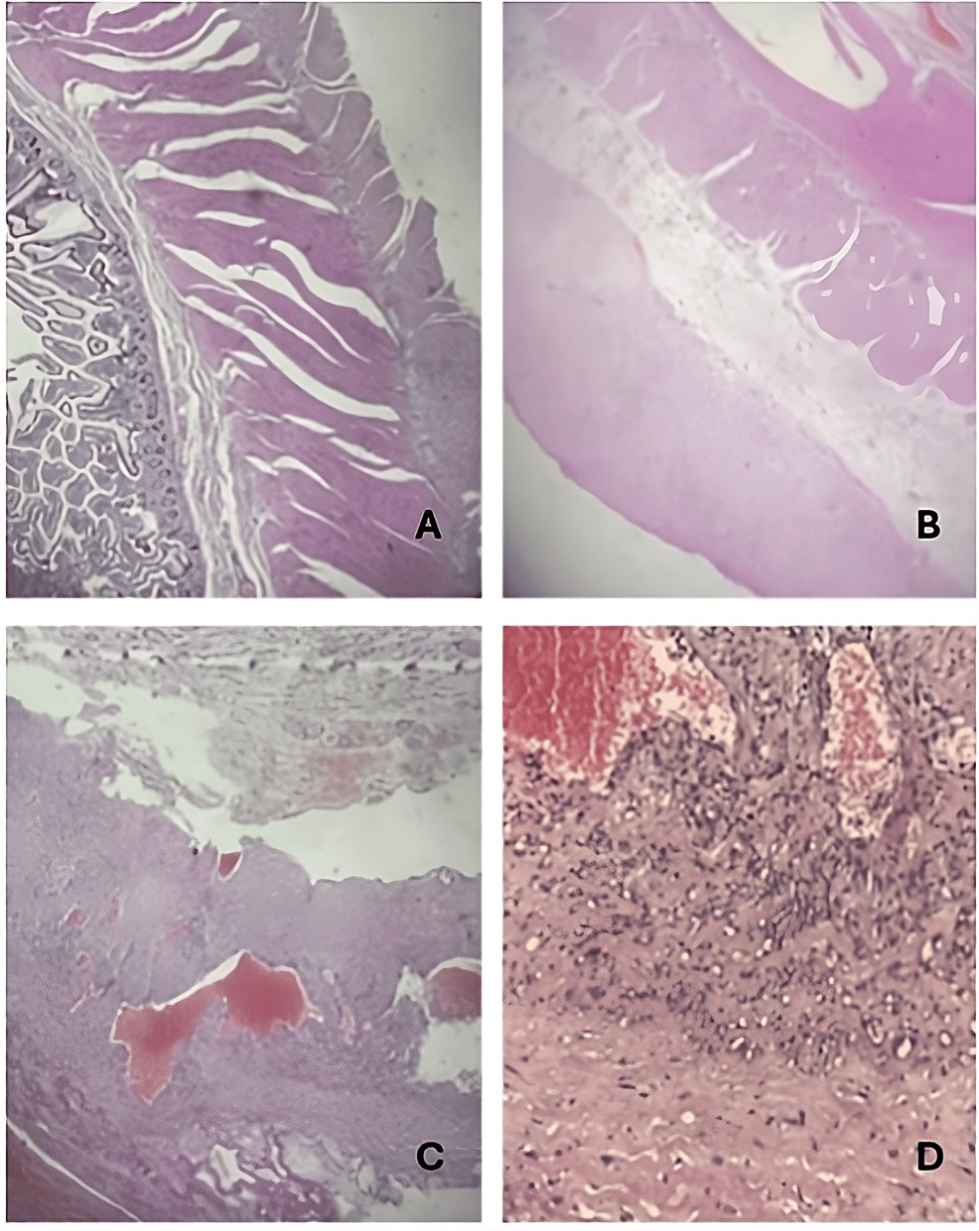
Histopathological study. A: Decrease in intestinal villi (200x) compatible with intestinal ischemia. B: Thickening of the peritoneum (200x) with an increase in the amount of connective tissue inside. C: Loss of intestinal villi (200x) associated with thrombosis of blood vessels. D: Chronic inflammatory infiltrate (100x), proliferation of fibroblasts, and abundant connective tissue.

In the postoperative period, the patient presented clinical deterioration associated with hepatic encephalopathy, hepatorenal syndrome, refractory hydroelectrolyte imbalance, and hemodynamic instability. After 48 hours, the patient died due to poor general conditions. The histopathological report confirmed the diagnosis of primary sclerosing peritonitis (Figure 5).

## Discussion

Intestinal obstruction is one of the most frequent surgical pathologies worldwide with a high rate of morbidity and mortality. Among the most frequent causes are adhesion syndrome, abdominal wall hernias, and gastrointestinal tumors. There is no prevalence or epidemiological data reported in our country or worldwide of CESP as an etiological factor, however, there are multiple reported cases where suggestions are made regarding the management of this disease.

CESP is a chronic inflammatory condition that causes peritoneal thickening due to the formation of a membrane of collagen and fibrous tissue that surrounds the loops of the small intestine. According to the studies carried out, CESP can be classified as primary (idiopathic) or secondary [3,4].

In idiopathic or primary CESP, hypoplasia of the greater omentum and malformation of mesenteric vessels have been discussed as risk factors. On the other hand, extensive endometriosis and the role of prostaglandin E2 and its relationship with the protease-activated receptor (PAR) have been described in women [4,5]. This can be classified into three categories based on the extension of the peritoneal membrane over the loops of intestine: type I refers to only partially covering a part of intestinal loops, type II covers the entire loops of the small intestine, and type III involves the entire loops of the small intestine and other organs (appendix, cecum, ascending colon, stomach, liver, and ovaries) [3].

Secondary CESP is related to chronic diseases or clinical conditions such as peritoneal dialysis, tuberculosis, sarcoidosis, ventriculoperitoneal valve status, systemic lupus erythematosus, liver cirrhosis, use of beta blockers, and chemotherapy, among others. However, it has been described that the most common correlation is with peritoneal dialysis [5]. The symptoms and clinical signs that patients may present are non-specific and may range from asymptomatic or symptoms of abdominal pain and intestinal obstruction with nausea and vomiting, anorexia, weight loss, or acute abdomen secondary to perforation, as in our patient [1,2].

The presurgical diagnosis of CESP is complex and requires high clinical suspicion. Generally, the diagnosis is made transoperatively, when a fibrous layer is found attached to and surrounding the intestinal loops. Currently, there is no specific clinical or radiological patterns to confirm the diagnosis [5]. However, computed tomography (CT) has proven to have the highest sensitivity and specificity for diagnosis as it can view thickening and calcification of the peritoneum associated with dilation of the intestinal loops. The “cauliflower sign” has been described, which consists of an agglomeration of intestinal loops by a thick membrane, characteristic of the disease [6]. A thickening greater than or equal to 2 mm appears to be an appropriate cut-off value in tomographic findings [7]. Other imaging findings that have been associated with the disease are loculated ascites and increased density of mesenteric fat. It is worth mentioning that a comparison of CT with MRI has been carried out, and it has been concluded that multidetector CT with multiplanar reformation is indeed the best diagnostic study, which is why it could have a transcendental role pre-surgically [8,9].

In our case, the patient presented with intestinal obstruction and acute abdomen in the same way as some previously reported cases [10,11]. The histopathological findings of a thickening of the fibroconnective tissue of the peritoneum, acute and chronic inflammatory infiltrates, lymphatic dilation without evidence of foreign body granulomas, giant cells, or birefringent material give the definitive diagnosis.

Medical treatment is mainly based on the use of oral corticosteroids and other immunosuppressants and drugs with anti-inflammatory effects such as mycophenolate mofetil, colchicine, or tamoxifen, which inhibit the inflammatory process of the peritoneal membrane and inhibit the synthesis and maturation of collagen [12,13]. These are offered to patients who have presented clinical symptoms of intestinal obstruction and imaging studies compatible with the disease. Surgical treatment is reserved for failures in medical treatment or as a need for surgical emergencies with intestinal obstruction and compromise of intestinal vitality. In cases where there is a clinical diagnosis of intestinal obstruction that doesn’t respond to medical management or with an indication of surgical urgency, or for cases when it is identified during surgery, an excision of the membrane with adhesiolysis must be performed. In cases where there is intestinal injury, resection and anastomosis with or without protective enterostomy is the treatment of choice. In cases where the membrane can be removed completely, the risk of recurrence is low [14,15]. In the case described, because the patient presented with signs of an acute abdomen, conservative treatment was not a therapeutic option.

## Conclusions

Chronic encapsulated sclerosing peritonitis is a very rare pathology and there is little information in the literature worldwide. However, the surgeon must always take this diagnostic possibility into account when faced with intestinal obstruction in a patient with a history of chronic liver failure.
